# Supplementary material for: Cloud BioLinux: pre-configured and on-demand bioinformatics computing for the genomics community
Source: BMC Bioinformatics. 2012 Mar 19;13:42. doi: 10.1186/1471-2105-13-42 (PMC3372431; doi:10.1186/1471-2105-13-42)
Supplement: Additional file 1 — Supplementary 1 Cloud BioLinux software documentation in the form of a mini, self-contained website. Users need to download and uncompress the .zip file, and open through a web browser the "index.html" file available on the main directory. (ZIP 1823 kb). [file 1471-2105-13-42-S1.ZIP › Cloud-BioLinux-Package-Documentation/docs/contml.html]

Bio-Linux Software Documentation Pages

Back to search form

## contml

|  |  |
| --- | --- |
| Name | contml |
| Description | **contml** is part of the PHYLIP package  Copyright 1986-2004 by the University of Washington. Written by Joseph Felsenstein. Permission is granted to copy this document provided that no fee is charged for it and that this copyright notice is not removed.  This program estimates phylogenies by the restricted maximum likelihood method based on the Brownian motion model. It is based on the model of Edwards and Cavalli-Sforza (1964; Cavalli-Sforza and Edwards, 1967). Gomberg (1966), Felsenstein (1973b, 1981c) and Thompson (1975) have done extensive further work leading to efficient algorithms. CONTML uses restricted maximum likelihood estimation (REML), which is the criterion used by Felsenstein (1973b). The actual algorithm is an iterative EM Algorithm (Dempster, Laird, and Rubin, 1977) which is guaranteed to always give increasing likelihoods. The algorithm is described in detail in a paper of mine (Felsenstein, 1981c), which you should definitely consult if you are going to use this program. Some simulation tests of it are given by Rohlf and Wooten (1988) and Kim and Burgman (1988).  **References:**  Felsenstein, J. 1993. PHYLIP (Phylogeny Inference Package) version 3.5c. Distributed by the author. Department of Genetics, University of Washington, Seattle.    Felsenstein, J. 1989. PHYLIP -- Phylogeny Inference Package (Version 3.2). Cladistics 5: 164-166. |
| Homepage | http://evolution.genetics.washington.edu/phylip.html |
| Remote Documentation | http://evolution.genetics.washington.edu/phylip/doc/contml.html |
